# Systematic review exploring the quality of life of patients undergoing mental disorders treatment in the kingdom of Saudi Arabia

**DOI:** 10.1186/s12991-026-00665-2

**Published:** 2026-05-24

**Authors:** Abdullah U. Althemery

**Affiliations:** Department of Clinical Pharmacy, College of Pharmacy, Prince Sattam Bin Abdulaziz University, 16278 Alkharj, Saudi Arabia

**Keywords:** Mental disorders, Quality of life, Psychotropic drugs, Patient-reported outcome measures, Mental disorders treatment

## Abstract

**Background:**

According to the World Health Organization, approximately one in eight people is diagnosed with mental disorders globally. Patient-reported outcomes and quality of life assessments are essential tools for evaluating patient outcomes. This study aimed to identify, characterize, and summarize the literature focusing on quality of life or patient-reported outcomes among patients with mental disorders in the Kingdom of Saudi Arabia undergoing pharmacological treatment.

**Methods:**

This systematic review was based on the Preferred Reporting Items for Systematic Reviews and Meta-Analyses 2020 item checklist applicable for Systematic reviews. Searches were run in the following databases: PubMed, MEDLINE via Ovid, MEDLINE via EBSCO, and ProQuest. Databases were searched from January 1, 2000, to July 31, 2025.

**Results:**

Ten articles that explored the quality of life of patients undergoing mental disorders treatment in the Kingdom of Saudi Arabia since 2000 met the inclusion criteria. A total of 3,773 patients included, and fifteen different quality of life questionnaires and patient-reported outcome measures were used. One-third of the reviewed articles used the Morisky Medication Adherence Scale (MMAS) and consistently reported low to moderate levels of adherence.

**Conclusions:**

This study highlights the need for further research in the mental health field, with a focus on enhancing patients’ quality of life and reporting outcomes.

**Supplementary Information:**

The online version contains supplementary material available at 10.1186/s12991-026-00665-2.

## Background

Mental disorders are prevalent worldwide and significantly affect individuals, families, and societies. According to the WHO, approximately one in eight people are diagnosed with mental disorders globally [1]. Mental treatment aims to alleviate symptoms of mental disorders and improve an individual’s overall well-being. Various effective mental treatments are available, including prescriptions and psychotherapy [2]. Untreated mental disorders can lead to significant impairments in social, occupational, and personal functioning and increase the risk of suicide and other adverse outcomes [3]. According to a study published in *The Lancet Psychiatry*, mental health treatments are associated with reduced mortality rates and improved overall health and economic outcomes [4]. Therefore, the timely and effective treatment of mental disorders is crucial for promoting mental health and well-being. 

Saudi Arabia offers a unique context for mental health care due to both systemic and cultural factors. KSA was among the early countries to adopt mental health policies and legislation, following WHO recommendations and providing inpatient care comparable to high-income countries. However, challenges persist with the availability of providers and services for outpatients and primary care facilities [5]. Culturally, ongoing stigma around mental health negatively affects diagnosis and treatment. These factors create a distinct experience for patients with mental disorders in KSA [6].

In this context, patient-reported outcomes (PROs) and quality of life (QoL) assessments are critical for evaluating pharmaceutical interventions [7]. These tools evaluate the patient’s physical, mental, and social well-being. Healthcare providers can better understand how patients view their illnesses and their influence on their lives by employing PROs and QoL [8]. This information can assist healthcare practitioners in tailoring treatment programs to meet patients’ individual needs, improve adherence to therapy, and optimize treatment outcomes [7]. Furthermore, QoL and PRO assessments can be helpful to endpoints in clinical trials, providing insights into patient experiences that traditional clinical endpoints may not provide [9].

The Kingdom of Saudi Arabia (KSA) has increased national attention to mental health research [5]. The Saudi National Health and Stress Survey has increased the number of studies on various mental diseases. However, most published work focused on epidemiological and service delivery rather than QoL associated with pharmacological treatments. 

There is an opportunity for pharmacists to play a more significant role in mental health research and patient treatment in KSA [5]. Pharmacists can offer valuable insights into the treatment of patients with mental health issues, including medication adherence and potential drug interactions. This study aimed to identify, characterize, and summarize the literature focusing on quality of life or patient-reported outcomes among patients with mental disorders in the Kingdom of Saudi Arabia undergoing pharmacological treatment. This review does not evaluate the role of pharmacy-led services but provides research opportunities for pharmacists by identifying gaps in previous research investigations of medications using PROs.

## Methods

### Search strategy

This systematic review was based on the Preferred Reporting Items for Systematic Reviews and Meta-Analyses (PRISMA) 2020 item checklist applicable to Systematic reviews [10]. A multistep process was utilized to finalize the systematic report using a systematic review tool to guide the research, which included the following: 1) Specified participants, interventions, comparators, outcomes, setting, and type (PICOST) based on the recommended criteria [11, 12]; 2) A search query created for PubMed; 3) Translated search query to EBSCO and ProQuest using the Polyglot Search tool [13]; 4) Collected articles and checked for duplicates [13]; 5) Screened title and abstract; 6) Applied final checklist (Additional File 1) for eligible articles; 7) Backward and forward citation analyses; 8) Screened new titles and abstracts for final inclusion; 9) Extraction of all needed data from the final list; and 10) Analysis of results.

### Selection criteria (PICOST)

Participants were defined as patients with mental disorders diagnosed by professionals based on the World Health Organization, Chapter V: Mental and Behavioral Disorders [14]. The interventions were Food and Drug Administration (FDA)- and non-FDA-approved medications utilized for various mental disorders guided by tertiary pharmacotherapy resources [15]. The focus was on pharmacological treatments, as this review was carried out from a pharmacist’s perspective. For comparison, all listed pharmacological treatments for mental disorders in the interventions were included in addition to cognitive therapy or placebo. The outcome measure was QoL using validated generic or disease-specific measurements of mental disorders [8, 16]. The settings were defined by all studies published in KSA or by one major city within KSA from 2000 until July 31, 2025 [17]. The study designs included randomized controlled trials, observational studies (all types), cohort (longitudinal) studies, case–control studies, before-after studies, cross-sectional studies, and surveys.

The exclusion criteria were studies with self-reported mental disorders that failed to mention the pharmaceutical treatment utilized, did not capture any QoL or PRS measurement, were published before 2000, or used data from outside Saudi Arabia. Articles in the press, conference abstracts, and books or book chapters were excluded from the search. Table 1 summarizes PICOST, and detailed definitions are provided in Additional File 2.

**Table 1:** Summary of PICOST definitions used to generate the search

Participants	Mental disorders
Interventions	FDA-approved and off-label treatments
Comparators	All medications listed in the interventions, cognitive therapy, placebo
Outcomes	Generic QoL measurements, PROs, and mental disorder disease-specific measures
Settings	The Kingdom of Saudi Arabia
Type	Randomized controlled trials, observational studies, cohort studies, case–control studies, before-after studies, cross-sectional studies, surveys

FDA: Food and Drug Administration, QoL: quality of life, PROs: patient-reported outcomes

### Search strings for bibliographic databases

**Fig. 1:**
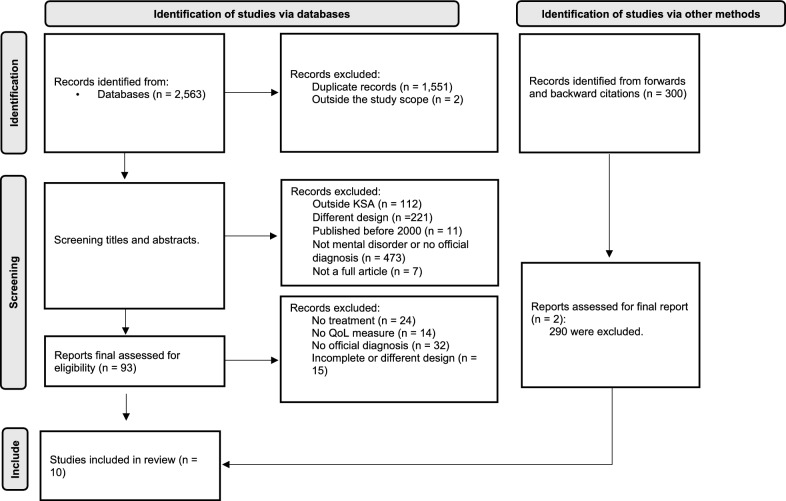
Detailed flow diagram for articles identification and screening

Searches were run in the following databases: PubMed, MEDLINE via Ovid, MEDLINE via EBSCO, and ProQuest. Databases were searched from January 1, 2000, to July 31, 2025. The search was restricted to only include English and Arabic. In addition to searching databases, backward and forward citation analyses were performed on the final articles. The detailed search process is illustrated in Fig. 1 using a modified PRISMA 2020 flow diagram. [10] Detailed definitions are provided in Additional File 2.

### Study screen and selection

Three independent reviewers (the principal author and two collaborators) screened the titles and abstracts of the articles. The principal author retrieved the eligible studies and checked the final criteria using a uniform checklist (Additional File 1). Any discrepancies were resolved by consensus. Automation tools have been used to help screen articles uniformly [11]. The studies were categorized based on title, year of publication, city (where data were collected), type of mental disorder studied, QoL measurement or PRO tool, number of patients included, and type of pharmaceutical treatment.

The study screening was conducted twice, initially in July 2023 and then in September 2025 to include recently published articles and for validation purposes. A final validation by an independent reviewer was conducted after the protocol was registered in the PROSPERO database. [18].

### Study analysis

The quality of the reviewed articles was assessed using the Newcastle–Ottawa Scale (NOS) for case–control and cohort studies. The NOS evaluates three domains of each article: selection (maximum for points), comparability (maximum two points), and exposure or outcomes (maximum three points). A final score ranging from 0–9 was summed for each article (Additional File 3). [19].

I applied automated word search criteria for the top 10 frequent words in the final studies to explore the research focus (Additional File 4). The results were merged for synonymous terms. Moreover, for validation, word frequencies were applied to exclude articles outside KSA before 2000, which were not related to mental disorders, and not entire articles. The top five words were reported for each category. [11].

## Results

The key characteristics of the ten included studies are summarized in Table 2. The publications starting from 2012 to 2024, with the highest number of studies (n = 3) published in 2018. Geographically, all studies were conducted within the Kingdom of Saudi Arabia (KSA), with eight studies including participants from Riyadh and two being multicenter studies conducted in various cities. The most frequently investigated mental disorders were depression (n = 4 studies, 40%) and general psychiatric conditions (n = 2 studies, 20%), followed by autism, obsessive–compulsive disorder (OCD), Kleine-Levin syndrome (KLS), and treatment-resistant depression (TRD)/bipolar/schizophrenia (n = 1 study each). The total sample size across all studies was 3,773 participants, ranging from a single case report to a multicenter study of 1,185 patients. All studies focused on pharmacological treatments, primarily antipsychotics, antidepressants, and other psychotropic medications.

**Table 2:** Characteristics of studies assessing quality of life and patient-reported outcomes among patients with mental disorders in Saudi Arabia

#	Final articles	Year	City	Type of mental disorder studied	# of patients	Treatment type
1	AlRuthia [20]	2024	Riyadh	TRD, bipolar disorder, and schizophrenia	100	antipsychotics
2	Alenezi et al. [21]	2022	Various cities in KSA	Autism	293	Psychotropic
3	Alatiq & Alrshoud [22]	2018	Riyadh	OCD	1	Not specified
4	Alosaimi et al. [23]	2018	Various cities in KSA	Psychiatric	1185	Antipsychotics and antidepressants
5	Al Shareef et al. [24]	2018	Riyadh	Kleine-Levin syndrome	10	All medications
6	Aljumah & Hassali [25]	2015	Riyadh	Depression	239	Antidepressants
7	Aljumah et al. [26]	2014	Riyadh	Depression	403	Antidepressant
8	Aljumah et al. [27]	2014	Riyadh	Depression	403	Antidepressant
9	Alhabeeb et al. [28]	2013	Riyadh	Depression	557	Psychiatric treatments
10	Alhabeeb et al. [29]	2012	Riyadh and Dammam	Psychiatric	899	Psychotropic drug

TRD: treatment-resistant depression, OCD: obsessive–compulsive disorder, KLS: Kleine–Levin syndrome

A total of fifteen different Patient-Reported Outcome (PRO) and Quality of Life (QoL) instruments were employed across the ten studies (Table 3) [20–29]. Three studies focusing on depression utilized the Morisky Medication Adherence Scale (MMAS), consistently reporting low to moderate levels of adherence (scores ranging from 3.06 to 7.68, mean: 5.21) [25–27]. The Beliefs about Medicines Questionnaire (BMQ), used in the same set of studies, identified that specific patient concerns about medication (e.g., perceived necessity versus harm) were significant barriers to adherence [25–27]. In contrast, the Treatment Satisfaction Questionnaire for Medication (TSQM) demonstrated that higher levels of treatment satisfaction were associated with more positive clinical outcomes.

**Table 3:** Quality of life and patient-reported outcome instruments used in the included studies

#	Instrument	Comment
1	Morisky medication adherence scale [25–27]	More patients were associated with lower adherence scores, with an average score of 5.21
2	Beliefs about medicine Questionnaire [25–27]	Specified concerns identified by the Beliefs about Medicine Questionnaire significantly impacted effectiveness and adherence
3	Research developed questionnaires [21, 23, 29]	Most surveys aim to collect patients’ characteristics, clinical information, and treatment utilization
4	Treatment satisfaction questionnaire for medication [24, 25]	Higher Satisfaction scores associated with positive outcomes
5	The Parental concerns questionnaire [21]	A disease-specific questionnaire developed for children with autism
6	The global severity index [22]	The authors measured patients’ self-reported symptoms with various methods. Cognitive intervention helps to minimize symptoms
7	The positive symptom total [22]	
8	The positive symptom distress index [22]	
9	the glasgow antipsychotics side effects scale	Two-thirds of the patients reported experiencing side effects, particularly for patients with comorbid conditions
10	Patient health questionnaire (PHQ-9)	Half of the study reported a score of moderate to severe depression
11	Stanford KLS questionnaire [24]	A disease-specific questionnaire with 280 questions – the results were comparable with international scores
12	The montgomery–åsberg depression rating scale [25]	Clinicians based questionnaire to measure depression severity; there was no significant difference for pharmacist consultation intervention on the scale score
13	The observing patient involvement in decision-making scale [25]	Measures patients’ involvement in the medical decision process. No results were reported
14	The EQ-5D [25]	One study used generic measurement. An average utility score of 0.67
15	the columbia suicide severity rating scale [28]	Clinicians-based scale for depression and suicidal communications

QoL: quality of life, PRO: patient-reported outcome, EQ-5D: EuroQol-5 Dimension, KLS: Kleine–Levin syndrome

Three studies have developed local context instruments to capture patient-reported outcomes in mental health care. Alenezi et al. [21] adapted the Parental Concerns Questionnaire (PCQ) for Arabic-speaking caregivers of children with autism to document psychotropic medication use among Saudi families [21]. Alosaimi and colleagues (2018) examined physical activity as a self-reported outcome in a large cross-sectional sample of 1,185 mental disorders patients across six hospitals, demonstrating significant associations between activity levels, psychiatric diagnoses, and psychotropic pharmacological regimens [23]. Alhabeeb et al. (2012) introduced structured outcome classification in child and adolescent psychiatric clinics by screening records from 899 patients, identifying risk factors such as perinatal complications and comorbidity that predicted unstable outcomes [29]. These original studies show how Saudi researchers have developed or adapted measurement strategies to generate culturally relevant patient-reported data that link treatment exposures with lived experience. However, direct treatment comparisons were generally not reported, and patient-reported outcomes were mainly used as descriptive assessments within treated populations.

Only one study employed a generic QoL instrument, the EQ-5D, reporting a mean utility score of 0.67, which indicates a substantially impaired quality of life relative to healthy population norms [25]. Disease-specific tools, such as the Stanford KLS questionnaire and the Parental Concerns Questionnaire for autism, were effectively used to identify the unique burdens associated with specific conditions [24]. The results derived from these disease-specific tools were generally comparable to international data.

Instruments such as the Montgomery-Åsberg Depression Rating Scale (MADRS) and the Global Severity Index were used to measure symptom severity. The evidence suggests that while pharmacological therapies led to improvements in clinician-rated symptoms, the corresponding gains in overall patient-reported quality of life were variable and appeared to be dependent on the specific mental disorder. [25].

The Newcastle Ottawa Scale scores for the included studies ranged from 4 to 8. Studies with higher quality scores had a clear methodological design and comparability across study groups. The highest-scoring study audited randomly selected medical records from well-established Ministry of Health child and adolescent psychiatric clinics and compared stable and unstable cases using multivariable logistic regression while adjusting for multiple sociodemographic and clinical characteristics [29]. Studies with lower quality scores were mainly descriptive in nature, with limited or no adjustment for confounding factors and no formal comparator groups. [22, 26].

## Discussion

This systematic review aimed to synthesize the evidence on quality of life (QoL) and patient-reported outcomes (PROs) among patients undergoing pharmacological treatment for mental disorders in the Kingdom of Saudi Arabia. My analysis of ten studies reveals a critically underdeveloped field of research, characterized by a scarcity of studies, a narrow geographical and methodological focus, and a predominant emphasis on medication adherence over holistic QoL assessment. The findings highlight significant gaps in patient-centered mental health research within KSA and underscore an urgent need for a more robust and diversified evidence base.

The most striking finding is the extreme paucity of relevant literature. Only ten studies over a 23-year period met the inclusion criteria, representing a mere fraction (0.634%) of the screened records. This stands in stark contrast to international systematic reviews on similar topics, such as the work by van Krugten et al. (2021), where a significantly higher proportion (16.57%) of screened articles were relevant [30]. This discrepancy underscores that while KSA has initiated national mental health efforts, patient-centered outcomes research specifically tied to pharmacological treatments lags considerably behind global standards. The concentration of studies in Riyadh, while understandable due to its robust healthcare infrastructure, limits the generalizability of the findings and highlights a significant geographic disparity in research capacity and patient access across the kingdom.

The synthesis of PRO instruments revealed a primary focus on medication adherence and beliefs (e.g., MMAS, BMQ). Low to moderate adherence, heavily influenced by medication concerns, align with global literature on chronic diseases [31]. However, the near absence of generic QoL measures like the EQ-5D, used in only one study. The reported mean utility score of 0.67 in that single study suggests a substantial QoL impairment, but without broader application, it is impossible to gauge the true burden of mental disorders on overall well-being in KSA or to conduct economic evaluations (e.g., cost-utility analyses) essential for informed healthcare policy [31, 32].

My findings both converge and diverge from international evidence. The effectiveness of disease-specific tools (e.g., for autism and Kleine-Levin syndrome) in identifying condition-specific burdens, with results comparable to international data, suggests that the manifestation of these disorders and their impact on PROs may be consistent across cultures. This reinforces the global relevance of these instruments.

However, the exploration of broader QoL domains contrasts with international trends, where the use of both generic and specific PROs is standard practice in mental health trials [33, 34]. Two surprising domains that were predominant within review articles were the family issues and suicidal attempts, even though not part of my original search strategy, resonate strongly with global evidence [33, 35]. It indirectly confirms that the consequences of mental illness extend beyond the individual to families and communities and highlights the well-established link between mental disorders, suicidality, and physical health comorbidities [33, 34, 36]. That these themes emerged organically from the data suggests that Saudi researchers are aware of these critical issues, even if they are not yet the primary focus of structured assessment [37].

The findings from this review carry distinct implications for key stakeholders: clinicians in KSA must integrate adherence counseling and address patient beliefs as a standard component of care to mitigate consistent medication non-adherence; researchers are urged to prioritize well-designed, nationally representative studies that employ both generic and disease-specific instruments to investigate broader outcomes like functional status and caregiver burden, moving beyond a narrow focus on adherence; and for policymakers, addressing the identified evidence gap is essential, requiring them to prioritize and fund patient-centered outcomes research to generate the necessary data for guiding resource allocation, shaping effective services, and conducting informed cost-effectiveness analyses.

This study lays the groundwork for future pharmacy-related research by being one of the first to examine the quality of life (QoL) of patients with mental disorders in Saudi Arabia from the viewpoint of a pharmacist. Nonetheless, it is important to recognize a number of limitations. First, only published publications that satisfied the specified eligibility requirements were included in the study; this could have resulted in selection bias and left out pertinent conference proceedings, current research, and gray literature. Second, only English-language papers were included in the search, which might have resulted in language bias and the exclusion of potentially pertinent research done in other languages. Third, because studies with negative or non-significant findings are less likely to be published, it is impossible to completely rule out the possibility of publication bias in the Saudi literature, as is the case with many reviews. Finally, the study did not include non-clinical groups such as students or community-based samples, despite the fact that omitted papers using phrases such as "students," "anxiety," and "COVID" indicate that significant research has been conducted in these areas. Future assessments should encompass these broader populations and specifically discuss methodological quality to get more complete results.

## Conclusions

This review found that, in Saudi Arabia, studies assessing quality of life and patient-reported outcomes in the pharmacological treatment of mental disorders are limited, and that patients, especially those with depression, commonly show low medication adherence and hold negative beliefs about treatment. To strengthen evidence and improve care, future research should expand geographically within the kingdom and use both generic and disease-specific QoL and PRO measures suitable for cost-utility analyses. Clinicians can improve outcomes by routinely integrating these measures into practice and addressing patient concerns to support adherence.

## Supplementary Information

Additional file 1.

Additional file 2.

Additional file 3.

Additional file 4.

## Data Availability

Available from the corresponding author upon reasonable request.

## Abbreviations

WHO: World health organization
PRO: Patient-reported outcome
QoL: Quality of life
KSA: Kingdom of Saudi Arabia
PRISMA: Preferred reporting items for systematic reviews and meta-analyses
PICOST: Participants, interventions, comparators, outcomes, setting, and type
FDA: Food and drug administration
NOC: The Newcastle–Ottawa scale
