# Supplementary material for: Systematic review exploring the quality of life of patients undergoing mental disorders treatment in the kingdom of Saudi Arabia
Source: Ann Gen Psychiatry. 2026 May 24;25:61. doi: 10.1186/s12991-026-00665-2 (PMC13383563; doi:10.1186/s12991-026-00665-2)
Supplement: Supplementary file 1 — Additional file 1. [file 12991_2026_665_MOESM1_ESM.docx]

**Additional File 1:** Query and final checklist

| In Saudi | Mental and behavior disorder | Treatment mentioned | QoL or PROs |
| --- | --- | --- | --- |
| ✓ | ✓ | ✓ | ✓ |

QoL: quality of life, PROs: patient-reported outcomes
