# Supplementary material for: Systematic review exploring the quality of life of patients undergoing mental disorders treatment in the kingdom of Saudi Arabia
Source: Ann Gen Psychiatry. 2026 May 24;25:61. doi: 10.1186/s12991-026-00665-2 (PMC13383563; doi:10.1186/s12991-026-00665-2)
Supplement: Supplementary file 2 — Additional file 2. [file 12991_2026_665_MOESM2_ESM.docx]

**Additional file 2:** Detailed PICOST MeSH terms used to generate the search

**PICOST**

| **PICOS** |  |
| --- | --- |
| **P**opulation | Patients with Mental disorders |
| **I**ntervention | Pharmacological treatments for Mental disorders |
| **C**omparison | Pharmacological treatments for Mental disorders or Cognitive therapy or placebo |
| **O**utcome | Quality of life using validated measure (generic and disease specific) |
| **S**etting | Institutes within the Kingdom of Saudi Arabia from 2000 inward |
| **S**tudy type | Quantitative studies |

| **Population** | **Search String** |
| --- | --- |
| Mental disorders | Mental disorders, Dementia, alcohol, substance use, substance abuse, substance addiction, Schizophrenia, delusional disorders, Manic, Bipolar, Depressive, Depression, psychoactive, sedatives, hypnotics, psychotic disorders, anxiety, Obsessive, compulsive, Somatoform, Eating disorders, sleep disorders, dependence, and Tic disorders |

| **Interventions** | **Search String** |
| --- | --- |
| Treatments for mental disorders | Pharmacological, treatments, therapy, Duloxetine, Venlafaxine XR, Antidepressants, Azapirone, Buspirone, Diphenylmethane, Hydroxyzine, Anticonvulsant, Pregabalin, Atypical antipsychotic, Lithium, Divalproex, Valproic acid, Valproate, Lamotrigine, Carbamazepine, Oxcarbazepine, antipsychotics, Aripiprazole, Asenapine, Cariprazine, Lurasidone, Olanzapine, Quetiapine, Risperidone, Ziprasidone, Selective serotonin reuptake inhibitors, SSRIs, Citalopram, Escitalopram, fluoxetine, Fluvoxamine, Paroxetine, Sertraline, Serotonin–norepinephrine reuptake inhibitors, SNRIs, Desvenlafaxine, Duloxetine, Levomilnacipran, Tricyclic antidepressants, TCAs, Amitriptyline, Desipramine, Doxepin, mipramine, Nortriptyline, Norepinephrine and dopamine reuptake inhibitor, NDRI, Bupropion, Mixed serotonergic effects, mixed 5­HT, Nefazodone, Trazodone, Vilazodone, Vortioxetine, Serotonin and α2 ­adrenergic antagonist, Mirtazapine, Monoamine oxidase inhibitors, MAOIs, Phenelzine, Selegiline, Tranylcypromine, Isocarboxazid, Brexpiprazole, Triiodothyronine, Naltrexone, Buprenorphine, Methadone, Chlorpromazine, Fluphenazine, Haloperidol, Loxapine, Loxapine inhaled, Perphenazine, Thioridazine, Thiothixene, Trifluoperazine, Aripiprazole, Asenapine, Brexpiprazole, Cariprazine, Clozapine, Iloperidone, Paliperidone, Suvorexant, Ramelteon, Valerian, Estazolam, Eszopiclone, Flurazepam, Quazepam, Temazepam, Triazolam, Zaleplon, Zolpidem, Multivitamin, Thiamine, Clonidine, Lorazepam, Chlordiazepoxide, Diazepam, Oxazepam, Dexmedetomidine, Phenobarbital |

| **Comparison** | **Search String** |
| --- | --- |
| Pharmacological treatments for Mental disorders or Cognitive therapy or placebo | - Other intervention listed within the intervention section. - Placebo - Cognitive therapy |

| **Outcome** | **Search String** |
| --- | --- |
| Quality of life using validated measure | - - - SF-36     - SF-12     - SF-6D     - PROMIS     - HRQoL     - Well-being Scale (QWB)     - WHOQOL     - Health Utilities Index (HUI)     - EQ-5D     - EuroQol     - Zung Self-Rating Depression Scale     - Cardiac Depression Scale     - Generalized Anxiety Disorder Scale     - Alcohol Use Disorders Identification Test     - Michigan Alcoholism Screening Test     - Depression Anxiety Stress Scales     - Beck Anxiety Inventory     - State-Trait Anxiety Inventory For Adults     - Anxiety, Depression And Mood Scale     - Mood And Anxiety Symptoms Questionnaire     - Revised Child Anxiety And Depression Scale     - Beck Depression Inventory     - Center For Epidemiological Studies-Depression Scale     - Depression And Family Functioning Scale     - Fatigue Associated With Depression Questionnaire     - Geriatric Depression Scale     - Patient Health Questionnaire     - Symptom Checklist-90-Revised     - Warwick Edinburgh Mental Wellbeing Scale     - Kessler 10 Psychological Distress Scale     - Profile Of Mood States     - Depression in patients with epilepsy     - Anxiety among patients with chronic obstructive pulmonary disease (COPD) |

| **Settings** | **Search String** |
| --- | --- |
| The Kingdom of Saudi Arabia | KSA, Saudi Arabia, and (largest cities adapted from the largest cities, by populations, identified from General authorities for statistics cities include: Riyadh, Makkah, Madinah, Qassim, Jeddah, and Dammam |

| **Study type** | **Search String** |
| --- | --- |
| Quantitative studies | - Primary source studies with quantitative analyses   - Clinical trials   - Cohort studies   - Case control   - Cross-sectional   - Case reports   - Case series) |
