# Supplementary material for: Systematic review exploring the quality of life of patients undergoing mental disorders treatment in the kingdom of Saudi Arabia
Source: Ann Gen Psychiatry. 2026 May 24;25:61. doi: 10.1186/s12991-026-00665-2 (PMC13383563; doi:10.1186/s12991-026-00665-2)
Supplement: Supplementary file 3 — Additional file 3. [file 12991_2026_665_MOESM3_ESM.docx]

**Additional File 3:** Newcastle–Ottawa Scale quality assessment

| **File** | **Study / Topic** | **Selection**  **(max 4)** | **Comparability**  **(max 2)** | **Outcome/Exposure**  **(max 3)** | **Total** |
| --- | --- | --- | --- | --- | --- |
| 1 | AlRuthia ^20^ | 3 | 2 (age, sex, comorbidities adjusted) | 2 | 7/9 |
| 2 | Alenezi et al. ^21^ | 3 | 1 (age + comorbidities in regression, but limited) | 2 | 6/9 |
| 3 | Alatiq & Alrshoud ^22^ | 2 | 0 (no group comparisons) | 2 | 4/9 |
| 4 | Alosaimi et al.^23^ | 3 | 1 (adjusted for one confounder only) | 2 | 6/9 |
| 5 | Al Shareef et al.^24^ | 4 | 0 (descriptive only, no comparison) | 2 | 6/9 |
| 6 | Aljumah & Hassali ^25^ | 3 | 1 (controlled for 1 variable, not multiple) | 2 | 6/9 |
| 7 | Aljumah et al.^26^ | 2 | 0 (no confounder control) | 2 | 4/9 |
| 8 | Al Jumah et al.^27^ | 3 | 1 (controlled for age only) | 2 | 6/9 |
| 9 | Al-Habeeb et al.^28^ | 3 | 1 (some subgroup analysis/1 confounder) | 2 | 6/9 |
| 10 | Al Habeeb et al.^29^ | 4 | 2 (multivariable adjustment, ≥2 factors) | 2 | 8/9 |
