# Supplementary material for: Systematic review exploring the quality of life of patients undergoing mental disorders treatment in the kingdom of Saudi Arabia
Source: Ann Gen Psychiatry. 2026 May 24;25:61. doi: 10.1186/s12991-026-00665-2 (PMC13383563; doi:10.1186/s12991-026-00665-2)
Supplement: Supplementary file 4 — Additional file 4. [file 12991_2026_665_MOESM4_ESM.docx]

**Additional File 4:** Word frequencies for included and excluded abstracts

| **Category** | **Rank** | **Word** | **Frequency** |
| --- | --- | --- | --- |
| **Included Studies** (n=9) | 1 | Disorders | 29 |
|  | 2 | Saudi | 28 |
|  | 3 | Patients | 27 |
|  | 4 | Family | 14 |
|  | 5 | Sleep | 13 |
| **Excluded Studies** |  |  |  |
| *Reason: Conducted outside KSA* | 1 | Saudi Arabia | 80 |
|  | 2 | Pakistan | 36 |
|  | 3 | China | 26 |
|  | 4 | United States | 18 |
|  | 5 | Egypt | 16 |
| *Reason: Different Study Design* | 1 | Study | 341 |
|  | 2 | Review | 237 |
|  | 3 | Methods | 112 |
|  | 4 | Qualitative | 83 |
|  | 5 | Development | 76 |
| *Reason: Not a Mental Disorder Focus* | 1 | Medical | 1518 |
|  | 2 | Covid | 1192 |
|  | 3 | Students | 1182 |
|  | 4 | Anxiety | 1098 |
|  | 5 | Care | 961 |
